# Cardiovascular and musculoskeletal health disorders associate with greater decreases in physical capability in older women

**DOI:** 10.1186/s12891-021-04056-4

**Published:** 2021-02-16

**Authors:** Juopperi Samuli, Sund Reijo, Rikkonen Toni, Kröger Heikki, Sirola Joonas

**Affiliations:** 1 Kuopio Musculoskeletal Research Unit (KMRU), Institute of Clinical Medicine, School of Medicine, University of Eastern Finland (UEF), Kuopio, Finland; 2 Department of Orthopedics, Traumatology and Hand surgery, Kuopio University Hospital, Kuopio, Finland

**Keywords:** Physical capability, Health disorder, Elderly, Chronic disease, Death

## Abstract

**Background:**

Good physical capability is an important part of healthy biological ageing. Several factors influencing physical capability have previously been reported. Long-term reports on physical capability and the onset of clinical disorders and chronic diseases are lacking. Decrease in physical capacity has been shown to increase mortality. This study focuses on the prevalence of chronic diseases. The primary objective of the study was to reveal the association between physical capability and morbidity. Secondary objectives included the validity of self-reported physical capability and the association between baseline physical capability and mortality.

**Methods:**

The OSTPRE (Kuopio Osteoporosis Risk Factor and Prevention Study) prospective cohort involved all women aged 47–56 years residing in the Kuopio Province, Finland in 1989. Follow-up questionnaires were mailed at five-year intervals. Physical capability questions were first presented in 1994. From these women, we included only completely physically capable subjects at our baseline, in 1994. Physical capability was evaluated with five scale self-reports at baseline and in 2014 as follows: completely physically capable, able to walk but not run, can walk up to 1000 m, can walk up to 100 m and temporarily severely incapable. The prevalences of selected chronic diseases, with a minimum prevalence of 10% in 2014, were compared with the change in self-reported physical capability. Additionally, associations between long-term mortality and baseline physical capability of the whole 1994 study population sample were examined with logistic regression. The correlation of self-reported physical capability with functional tests was studied cross-sectionally at the baseline for a random subsample.

**Results:**

Our study population consisted of 6219 Finnish women with a mean baseline age of 57.0 years. Self-reported physical capability showed statistically significant correlation with functional tests. Cardiovascular diseases and musculoskeletal disorders show the greatest correlation with decrease of physical capability. Prevalence of hypertension increased from 48.7% in the full physical capability group to 74.5% in the “able to walk up to 100 metres” group (*p* < 0.001). Rheumatoid arthritis showed a similar increase from 2.1 to 7.4% between these groups. Higher baseline body mass index (BMI) decreases long-term capability (*P* < 0.001). Women reporting full physical capability at baseline had a mortality rate of 15.1%, in comparison to 48.5% in women within the “able to walk up to 100 m” group (*p* = 0.357). Mortality increased steadily with worsening baseline physical capability.

**Conclusions:**

The results of this study show that chronic diseases, particularly cardiovascular and musculoskeletal disorders, correlate with faster degradation of physical capability in the elderly. Similar results are shown for increase in BMI. We also demonstrate that the risk of mortality over a 20-year period is higher in individuals with poor baseline physical capability.

## Background

Physical capability is part of healthy ageing. Health is a broad concept, which includes biological, psychological and social health. As such, factors promoting healthy ageing must be approached from a multitude of viewpoints, one of which is healthy biological ageing. Research on healthy ageing lacks an agreed conceptual framework [1]. Healthy biological ageing can be characterized using the concept of physical capability. Physical capability refers to the ability to move about and carry out everyday tasks, and is an important part of daily life [2].

Physical capability can be measured objectively by using tests that measure physical capacity, such as grip strength, walking speed and chair rising. Capability can also be measured subjectively using self-reported data. Physical capacity tests are made in a controlled environment and reflect capability. The results of these tests have been shown to correlate with long-term mortality in the elderly, with follow-up times ranging from 5 to 20 years [3].

Good physical capability is a prerequisite of being physically active. Overall physical activity has been shown to have associations with health outcomes including all-cause mortality, cardiovascular disease, hypertension, type 2 diabetes, osteoporosis and colon and breast cancer [4]. Lack of physical activity has been shown to cause rapid maladaptation and loss in the total and quality of years of life. Lack of activity is also a major risk factor for diabetes and ischemic heart disease [4, 5]. Physical activity prevents or delays chronic diseases [6].

Physical capability is also reduced by the phenomenon of age-related sarcopenia. Physical activity and a protein-rich diet remain the best-known methods of deterring sarcopenia. Efforts to reduce the loss of muscle by increasing dietary protein intake without exercise or by using stand-alone pharmaceutical treatments have been disappointing [7, 8]. The development of physical capability has been shown to depend on a variety of factors in addition to age-induced effects. Factors such as socioeconomic status in childhood and factors in utero or early postnatal life have been shown to associate with later physical capability. The development of physical capability is a lifelong process and is complex in nature [1]. Previous reports on physical capability and the onset of clinical disorders and chronic diseases are sparse. The relationship between the development of physical capability and the effects of chronic diseases is largely unstudied. In this work we examined the long-term development of self-reported physical capability and underlying factors behind its deterioration.

Life expectancy is increasing globally [9] and physical capability is essential to daily life [2]. Those who suffer from limited physical capability are often reliant on others for the completion of their daily tasks. As the average life expectancy of individuals increases with each generation it is essential, from both a quality of life and societal cost viewpoint, to study the underlying factors leading to the deterioration of physical capability.

This study focuses on the prevalence of chronic diseases. The primary objective of the study was to reveal the association between physical capability and morbidity. Secondary objectives included the validity of self-reported physical capability and the association between baseline physical capability and mortality.

## Methods

### Osteoporosis risk factor and prevention study

**Fig. 1:**
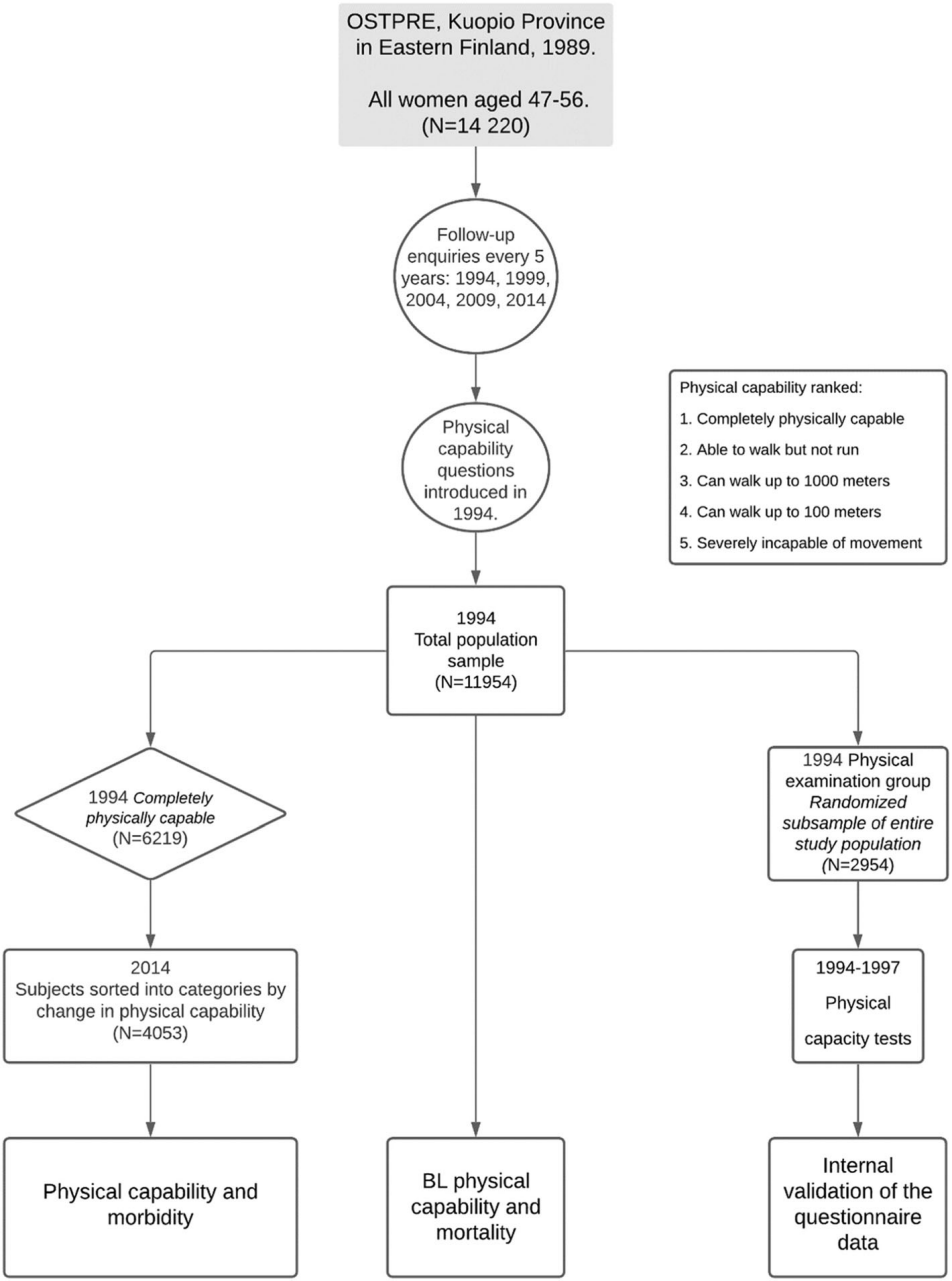
flowchart detailing the study population, exclusion and inclusion criteria and methods

This study was based on data collected from the “Kuopio Osteoporosis Risk Factor and Prevention” (OSTPRE) cohort [10]. The population-based study originally consisted of all women aged 47 to 56 who resided in the Kuopio province of Eastern Finland in April 1989. Questionnaires pertaining to the subjects’ current state of health and general lifestyle were sent by mail. Follow-up questionnaires were mailed at five-year intervals, i.e. 1994, 1999, 2004, 2009 and 2014. Questions pertaining to physical capability first appeared in the 1994 questionnaire, and we use this as our baseline (Fig. 1).

To study factors affecting the deterioration of capability, we included every respondent who had reported complete physical capability in the 1994 questionnaire as our baseline group. All other participants were excluded. We also contrasted baseline physical capability with overall mortality in the cohort, for which we included every respondent. Physical capacity tests were performed for a random subsample of the whole study population starting in 1994. Specially trained study nurses and a research secretary collected and recorded all data. We use this data to validate the answers given in the questionnaire. Due to limited resources to perform these measurements, physical capacity tests were not performed by all the participants. The randomisation process has been described earlier in detail [11]. Variance analysis was performed to examine differences between the whole study sample and the capacity test group.

The primary objective of the current study was to examine the association between physical capability and morbidity. Secondary objectives included the validity of self-reported physical capability and the association between physical capability and mortality. The study was approved by the Kuopio university hospital ethics committee on October 28, 1986 and was performed in accordance with the ethical standards set by the Declaration of Helsinki. All participants gave informed consent to their participation in the study.

### Self-reported physical capability

In order to compare and to contrast changes in self-reported physical capability, we collected responses to the 1994 and 2014 questionnaires. Both questionnaires consisted of largely similar pre-prepared categories for physical capability. In the 1994 questionnaire, participants were asked to rank their physical capability by selecting one of six answers (1. completely physically capable, 2. able to walk but not run, 3. can walk up to 1000 m, 4. can walk up to 100 m, 5. temporarily physically incapable of movement and 6. physically incapable of movement) and in the 2014 questionnaire physical capability was ranked by selecting one of seven answers (1. completely physically capable, 2. able to walk but not run, 3. can walk up to 1000 m, 4. can walk up to 100 m, 5. can only move indoors, 6. physically incapable of movement and 7. temporarily physically incapable of movement). The first four categories were uniform in both questionnaires and are represented unaltered in this study. The other categories had minor differences between them and in order to obtain comparable data, we combined the last categories as follows. We formed a fifth category, labelled “temporarily severely incapable”, by combining the two 1994 answers “temporarily physically incapable of movement” and “physically incapable of movement”. In the 2014 questionnaire we combined the answers “can only move indoors”, “physically incapable of movement” and “temporarily physically incapable of movement”. The answers were harmonized into a five-step ranking system for the purposes of the present study:

1. Completely physically capable
2. Able to walk but not run
3. Can walk up to 1000 m
4. Can walk up to 100 m
5. Temporarily severely incapable

### Chronic diseases and other morbidities

Participants were asked to report their physician diagnosed chronic diseases in the 1994 and 2014 questionnaires by selecting them from a pre-prepared list. To investigate the effect of chronic diseases we excluded all baseline participants who did not report themselves to be completely physically capable in 1994. Diseases with a minimum overall prevalence of 10% in the 2014 data pool were included. From these diseases we left out those that were considered not to be clinically relevant to physical capability, such as ocular diseases and allergies. Multimorbidity was measured as the total number of self-reported chronic diseases, whether they met the 10% selection criteria or not.

Musculoskeletal diseases fitting the prevalence and selection criteria were rheumatoid arthritis as well as osteoarthritis of the knees and hips. General back pain disorders were also included. It has previously been shown that arthritis decreases physical capability [12]. We categorized all different forms of back pain into one state collectively referred to as back pain. Cardiovascular diseases meeting the selection criteria were hypertension, coronary artery disease (CAD) and “other cardiovascular disease” denoting any heart condition not specified in the premade list.

Other self-reported chronic diseases meeting the selection criteria were diabetes, asthma and hypothyroidism. All types of diabetes (types 1 and 2) were included and are treated as a single group in order to investigate the effects of metabolic diseases on physical capability. Diabetes mellitus has previously been shown to reduce results in walking tests [13], and is associated with obesity. Bronchial asthma has been shown to reduce physical activity in adults [14]. Height and weight data were collected from the 1994 questionnaire. BMI was calculated as weight in kilograms divided by height in metres squared. Changes in BMI were also contrasted with the prevalence of the diseases studied.

### Correlation of self-reported physical capability with functional tests

In order to study the correlation between self-reported physical capability and functional measures of physical capability, we compared the questionnaire data to functional measurement data. In the baseline of the present study (1994), functional capacity tests were introduced for a random subsection of participants (*N* = 2954). Due to limited resources, these tests were not available to all participants. This randomization produced a population with a largely similar prevalence of baseline morbidities. The functional measurements examined for this study consisted of grip strength, knee extension force, being able to squat down to the floor and a balance test consisting of standing on one foot for 10 s without falling over. Grip strength was measured with a hand-held dynamometer by taking the mean of three successive attempts (Martin Vigorimeter, Tuttlingen, Germany). Knee extension force was measured three times from both legs (Dynamometer chair; Metitur Oy, Jyväskylä, Finland). The functional tests were performed in a controlled environment by trained research nurses. The data collected from these tests was compared cross-sectionally with the self-reported physical capabilities from the same year.

### Statistical methods

Sample size calculations were not made, because our cohort included all women aged 47–56 who resided in the Kuopio Province, Finland in 1989. Type I error was set to 0.05. The *p*-values for the prevalence of different chronic diseases in different categories of deterioration were calculated using chi-squared tests (χ^2^). The *p-*values for the association between categories of deterioration and 1) the mean number of chronic diseases and 2) baseline BMI were calculated using analysis of variance (ANOVA). Correlation between self-reported and measured physical capability was calculated using chi-squared tests (χ^2^) for bivariate data and analysis of variance (ANOVA) for grip strength and leg extension force. Odds ratio for mortality (overall cause of death) was studied with a bivariate logistic regression model using data collected from the Statistics Finland, Death and Cause of Death registry.

All statistical analyses were performed with SPSS software version 25 for Windows (IBM Corp., Armonk, NY).

## Results

### Characteristics

In 1989 all 14,220 women aged 47 to 56 residing in Kuopio were mailed questionnaires, with a total respondent number of 13,100 (92.8%). The response rate varied from 80 to 93% throughout the 25-year study, reported earlier in detail [15].

The OSTPRE cohort in 1994 consisted of 11,954 women with a mean baseline age of 57.3 (SD 2.9). Of these, 6219 women reported themselves to be completely physically capable in 1994. This group had a mean baseline age of 57.0 years (SD 2.9). Of these women a further 4053 answered the 2014 questionnaire. The randomized sample of 2954 women that underwent functional capacity tests had a mean baseline age of 59.1. (SD 2.9) (Table 1) (Fig. 1). Variance analysis showed no statistically relevant difference between the whole study sample and the capacity test group apart from baseline age and total number of chronic diseases.

**Table 1:** Baseline characteristics of the physical examination group, the total study population and the completely physically capable group in 1994. Analysis of variance between the total population sample and the physical examination group are reported as the *p*-value

Characteristic	Completely physically capable 1994 *N* = 6219	Total population sample *N* = 11,954	Physical examination group *N* = 2954	*p-*value
Age, years (SD)	57.0 (2.9)	57.3 (2.9)	59.1 (2.9)	< 0.01
Height, cm (SD)	161.3 (5.2)	161.2 (5.3)	160.0 (5.5)	0.659
Weight, kg (SD)	68.0 (10.6)	70.6 (12.2)	72.0 (18.5)	0.058
BMI (SD)	26.1 (3.8)	27.1 (4.5)	28.1 (4.9)	0.036
Mean of number of baseline chronic diseases per person (SD)	2.25 (1.404)	1.53 (1.358)	1.76 (1.340)	< 0.01
Smokers, %	13.5% (344)	14.8% (1159)	15.5% (310)	0.161
Average daily cigarettes	11.10	10.86	10.72	0.613
Average alcohol consumption (units of 12 g 100% alcohol per month)	61.3	63.7	66.1	0.476
Grip strength, kPa (SD)	N/A	N/A	71.2 (18.5)	N/A
Knee extension force, Kg (SD)	N/A	N/A	44.0 (11.9)	N/A
Able to squat to the floor, %	N/A	N/A	(1958/ 2610) = 75.0%	N/A
Able to stand on one foot for 10 s, %	N/A	N/A	(2099 / 2809) = 74.7%	N/A

### Correlation of self-reported physical capability with functional tests

Validation of the self-reported physical capability data was made by cross-checking the results of physical capacity measurements performed starting in 1994 and the self-reported levels of physical capability at the time of testing (Table 2). Those who reported themselves to have better physical capability outperformed on average those whose self-reported physical capability was poorer. This trend was evident in leg extension force, as well as in grip strength. Dichotomous tests measuring the ability to squat and perform standing balance tests also showed a gradual decrease in percentage with lower self-reported capability. Each of these tests showed a statistically significant correlation (*p* < 0.001) with higher self-reported physical capability and greater physical capacity. These trends did not apply to the “temporarily severely incapable” category.

**Table 2:** Association of self-reported physical capability with physical capacity tests in 1994. *N =* 2954

Physical capability (1994)	Completely physically cabable *N =* 1471	Able to walk but not run *N* = 993	Able to walk up to 1000 m *N* = 192	Able to walk up to 100 m *N* = 28	Temporarily severely incapable *N* = 22	*P*-value
Years between received postal data and measurements. Min – max mean (SD)	0.51–3.9	0.52–3.49	0.53–3.38	0.74–3.10	0.64–3.22	
	1.92 (0.76)	2.02 (0.75)	2.06 (0.75)	2.00 (0.69)	2.08 (0.71)	
Able to squat down to the floor, N (%)	1243 (85.9)	614 (64.8)	87 (48.9)	3 (16.7)	11 (57.9)	< 0.001
Able to stand on one foot for 10 s, N (%)	1402 (97.3)	893 (91.7)	143 (76.9)	9 (37.5)	18 (81.8)	< 0.001
Mean knee extension force, kg (SD)	35.49 (0.147)	33.22 (0.237)	30.591 (1.190)	25.273 (12.444)	33.214 (9.777)	< 0.001
Mean grip strength in dominant arm, kPa (SD)	74.901 (16.9)	67.704 (18.9)	66.542 (21.3)	57.571 (20.5)	67.818 (27.1)	< 0.001

### Physical capability and morbidity

The relationships between the prevalence of the studied diseases (osteoarthrosis of the knees, osteoarthrosis of the hips, coronary heart disease, other cardiovascular disease, hypertension, rheumatoid arthritis, diabetes (all types), back pain, asthma, hypothyroidism), the mean of number of chronic diseases per person and the mean of baseline BMI of the subjects and the development of self-reported physical capability are presented in Table 3.

**Table 3:** The prevalence and number of chronic diseases and baseline BMI in 2014 of the study population that were completely physically capable in 1994

Category, capability in 2014	Completely physically capable. *N* = 2046	Able to walk, but not run *N* = 1402	Able to walk up to 1000 m *N* = 368	Able to walk up to 100 m *N* = 148	Temporarily severely incapable *N* = 88	*p*-value
Mean of number of chronic diseases per person (SD)	4.79 (0.006)	6.71 (0.009)	8.45 (0.033)	8.14 (0.082)	6.35 (0.139)	< 0.001
Mean of baseline BMI (SD)	25.0 (0.06)	26.4 (0.01)	27.5 (0.03)	28.9 (0.09)	27.1 (0.14)	< 0.001
Osteoarthrosis of the knees, N (%)	345 (16.9)	468 (33.4)	138 (37.5)	50 (33.6)	26 (30.0)	< 0.001
Osteoarthrosis of the hips, N (%)	121 (5.9)	191 (13.6)	54 (14.7)	23 (15.4)	17 (19.3)	< 0.01
Coronary heart disease, N (%)	195 (9.5)	210 (15.0)	86 (23.4)	40 (26.8)	13 (14.8)	< 0.001
Other cardiovascular disease, N (%)	160 (7.8)	207 (14.8)	73 (19.8)	35 (23.5)	18 (20.5)	< 0.001
Hypertension, N (%)	996 (48.7)	875 (62.4)	241 (65.5)	111 (74.5)	55 (62.5)	< 0.001
Rheumatoid arthritis, N (%)	43 (2.1)	39 (2.8)	27 (7.3)	11 (7.4)	9 (10.2)	< 0.001
Diabetes (all types), N (%)	218 (10.7)	218 (15.5)	91 (24.7)	44 (29.5)	17 (19.3)	< 0.001
Back pain, N (%)	265 (13.0)	315 (22.5)	123 (33.4)	54 (36.2)	30 (34.1)	< 0.001
Asthma, N (%)	165 (8.1)	182 (13.0)	54 (14.7)	28 (18.8)	5 (17.2)	< 0.001
Hypothyroidism, N (%)	395 (19.3)	288 (20.5)	77 (20.9)	38 (25.5)	19 (21.6)	0.405

Subjects with the poorest end-point physical capability showed the greatest prevalence of diseases, ranging from 7.4% for Rheumatoid arthritis to 74.5% for hypertension (*p* < 0.001) in the “able to walk up to 100 metres.” group. Disease prevalence increased steadily with decreasing physical capability, except in the combined fifth category (temporarily severely incapable). Participants who were included in the highest physical capability group (i.e. experienced no reduction in self-reported physical capability) showed increases in the prevalence of the selected diseases only in the range of 3–5% during the 20-year follow up. The only exception to this trend was hypertension. The prevalence of hypertension increased by 30% between 1994 and 2004 among those who did not experience deterioration in their physical capability (*p* < 0.001).

At the 20-year follow-up, hypertension showed the greatest overall prevalence, with 48.7% in those who reported full physical capability in contrast to 74.5% in those who could only walk up to 100 m. A similar increase in prevalence was observed with back pain, with an increase from 13.0% (full physical capability) to 36.2% (could only walk up to 100 m). Coronary heart disease showed an increase from 9.5% (full physical capability) in contrast to 26.8% (could only walk up to 100 m) (*p* < 0.001). For other morbidities, similar increases between the “full physical capability” group and “could only walk up to 100 m” group were: asthma (an increase of 10.7%), osteoarthritis of the knees (13.1%), osteoarthritis of the hips (19.5%), coronary heart disease (17.3%) and other cardiovascular disease (15.7%) (*p <* 0.001). Hypothyroidism showed an increase in prevalence of 6.2%, but no statistically relevant correlation was observed. Baseline BMI was 25.0 kg/m^2^ in those who reported no change in their physical capability and 28.9 kg/m^2^ in those who could only walk up to 100 m at the end point. Increase in BMI between categories was gradual. Changes in BMI between baseline and endpoint showed no statistically significant correlation with the prevalence of the chronic diseases examined. This change was examined in the overall study population.

### Physical capability and mortality

The mortality of the total population sample (*N* = 11,149) was studied according to baseline physical capability with logistic regression (Table 4). Higher baseline physical capability was associated with lower odds of death. Those who were completely physically capable had lower odds of death (OR 0.240 95% CI 0.153–0.377 (*p* < 0.001)). Baseline capability had the highest effect on mortality, followed by the total number of chronic diseases and baseline age. Baseline BMI showed the least impact. Overall mortality in the study group was 18.4%, with a mortality percentage of 49.4% in those who reported themselves to be temporarily severely incapable.

**Table 4:** Odds-ratios of death associated with baseline physical capability, age, BMI and total number of chronic diseases. *N =* 11,149

Physical capability level in 1994:	Mortality. N (%)	OR for death	Confidence interval.	*p -*value
Completely physically capable (*N =* 6219)	15.1 (939)	1	ref	< 0.01
Able to walk but not run (*N* = 3918)	19.7 (770)	1.087	0.919–1.285	0.330
Can walk up to 1000 m (*N* = 758)	28.4 (215)	1.680	1.269–2.224	< 0.001
Can walk up to 100 m(*N* = 167)	48.5 (81)	3.469	2.060–5.843	0.357
Temporarily severely incapable (*N* = 87)	49.4 (43)	3.821	2.035–7.177	< 0.01
Baseline Age (*N =* 11,149)	N/A	1.103	1.076–1.131	< 0.01
Baseline BMI (*N =* 11,149)	N/A	1.014		0.088
Total number of chronic diseases at baseline (*N =* 11,149)	N/A	1.164	1.101–1.231	< 0.01
Alcohol consumption (increase of 1 unit of 12 g 100% alcohol per month)	N/A	1.000	0.999–1.000	0.237
Smoker	N/A	1.244	1.020–1.516	0.031

## Discussion

In this 20-year follow-up study we examined postal inquiry data collected from the OSTPRE cohort consisting of 14,220 women, starting in 1989. We set our baseline at the 5-year follow-up in 1994. From this group, we excluded all women who reported themselves not to be completely physically capable. Our selected study pool consisted of 6219 completely physically capable women with an initial mean age of 57.0 years. We examined the relationship between the prevalence of specific diseases and the deterioration rate of physical capability in this group by contrasting data collected in 2014. We also accounted for the effect of baseline BMI over the course of 20 years. In addition, we examined the effect of physical capability on mortality by contrasting baseline physical capability with mortality for all 11,954 women. We also examined the validity of self-reported physical capability by contrasting these results to a random subsample of the entire cohort who underwent physical capacity tests. Our results indicate that subjects whose physical capability deteriorate the fastest have a higher endpoint prevalence of chronic disease, particularly cardiovascular disease and musculoskeletal disorders. We also demonstrate that higher baseline capability reduces mortality.

The results of this study are largely in line with previous findings regarding physical activity and overall health. Our results show that a decrease in physical capability is associated with increasing mortality. Similar findings have been reported by other authors [7, 10]. Previously, physical activity has been shown both to reduce the onset of as well as to alleviate chronic disease [6]. Physical activity has previously been linked with several health benefits as well as with an increase in life quality and expectancy [16, 17].

This study is unique in that previous findings have not focused on the long-term development of physical capability. The relationship between chronic diseases and deterioration has been unclear. Our results indicate that physical capability is best maintained in subjects with the lowest numbers of chronic diseases. This study demonstrates a trend of higher prevalence of overall disease with deterioration of physical capability, the highest associations being with musculoskeletal and cardiac disorders. These results are in agreement with earlier findings, as lack of exercise has been shown to be a risk factor for diseases of both the cardiovascular and musculoskeletal systems [6]. Hypertension and cardiovascular disease are known to increase mortality [18].

The strength of the current study lies in its large population sample, high response rate and long follow-up time. Factors such as diet and exercise were outside the scope of the current study. Our large sample sizes give statistical strength to our findings. However, issues such as variable response rates to specific questions cause minor fluctuations in the availability of data, and the subjective nature of the questionnaires limits their accuracy. These issues are to some extent present in all questionnaire studies, but are mostly overshadowed by our high response rates. Our remodelling created a fifth category, labeled “temporarily severely incapable”, that does not align with the general trend of higher prevalence of disease and worsening physical capability. This category also produced anomalous results when contrasted with the physical capacity data collected between 1994 and 1997. We remodelled those who reported themselves to be temporarily incapable of movement into this category. The vagueness of this answer resulted in a rather heterogeneous fifth category. Details concerning the nature of the temporary disability were not available. In addition to this, the subjects who reported themselves to be temporarily severely incapable of movement still visited the capacity measurements and were able to perform the assigned tests. As such, this extreme of the categories should be viewed with some caution. In addition to this, part of the issue arises from the interpretations of our subjects of what it means to be “completely physically incapable”. However, this group was small in comparison to the total subject pool and as such does not skew the data in any significant way. Similar issues arise with the nature of self-reported diseases. Recall bias in observational studies cannot be totally excluded. This could be accomplished by contrasting the reports with registry data. Such data was not available for this study.

The physical capability scale used in the present study is not a validated scale. Nevertheless, from a clinical standpoint, it may be considered feasible to estimate the capability of movement. The scale provided also correlates well with the data collected from the functional tests. This finding is however limited by our limitation in measurement resources. Functional tests were available only for a randomly selected subsample of 2954 women.

Identifying the factors affecting physical capability is paramount in finding ways to uphold and develop it in an ageing population. Upkeep of physical capability is important from both an economic standpoint and from a quality-of-life perspective. The specifics of the relationship between the deterioration of physical capability and chronic disease remain largely unknown. Further research on the development of physical capability is warranted. Chronic disease may lead to deteriorating physical capability, or decreases in physical capability can lead to the development of disease. The specifics of this relationship are outside the bounds of this study. Chronic diseases develop over time, and as such the time of diagnosis of a disease cannot be used as its starting point. Claims of causality between the development of a disease and its effects on capacity are therefore difficult, if not impossible, to confirm.

## Conclusions

We have highlighted that chronic diseases have a role in the development of physical capability. In this 20-year follow-up we show that the prevalence of the studied diseases increases across the board with faster deterioration. The greatest deterioration was associated with cardiovascular and musculoskeletal disorders. We also show that low physical capability is associated with an increase in mortality rate. Preventative measures aimed at these diseases could help reduce the deterioration of physical capability, and therefore mortality.

## Data Availability

The datasets generated and/or analysed during the current study are available from the corresponding author on reasonable request.
