# Correction to: Cardiovascular and musculoskeletal health disorders associate with greater decreases in physical capability in older women

**DOI:** 10.1186/s12891-021-04219-3

**Published:** 2021-04-12

**Authors:** Samuli Juopperi, Reijo Sund, Toni Rikkonen, Heikki Kröger, Joonas Sirola

**Affiliations:** 1 Kuopio Musculoskeletal Research Unit (KMRU), Institute of Clinical Medicine, School of Medicine, University of Eastern Finland (UEF), Kuopio, Finland; 2 Department of Orthopedics, Traumatology and Hand surgery, Kuopio University Hospital, Kuopio, Finland

**Correction to: BMC Musculoskelet Disord 22, 192 (2021)**

**https://doi.org/10.1186/s12891-021-04056-4**

Following the publication of the original article [1] the authors noticed that the authors’ first name and last name were interchanged. The published version reads “Juopperi Samuli, Sund Reijo, Rikkonen Toni, Kröger Heikki and Sirola Joonas” which should be changed to “Samuli Juopperi, Reijo Sund, Toni Rikkonen, Heikki Kröger and Joonas Sirola”.

The original article [1] has been updated.
